# Identification of four differentially methylated genes as prognostic signatures for stage I lung adenocarcinoma

**DOI:** 10.1186/s12935-018-0547-6

**Published:** 2018-04-19

**Authors:** Wei-Ming Luo, Zheng-Yu Wang, Xin Zhang

**Affiliations:** 1 Department of Radiation Oncology, Shanghai Minhang District Cancer Hospital, 106 Ruili Road, Shanghai, 200240 China; 2 Department of Pharmacy, The Affiliated Huai’an Hospital of Xuzhou Medical University and The Second People’s Hospital of Huai’an, 62 South Huai’hai Rode, Huai’an, China; 3 Department of Medical Imaging, The Fourth People’s Hospital of Huai’an, Huai’an, Jiangsu China

**Keywords:** Lung adenocarcinoma, Prognostic signature, DNA methylation, Survival analysis

## Abstract

**Background:**

Lung adenocarcinoma (LUAD) is the main subtype of non-small cell lung cancer with a low survival prognosis. We aimed to generate a prognostic model for the postoperative recurrence of LUAD.

**Methods:**

The methylated DNA data of LUAD patients were downloaded from the Cancer Genome Atlas (TCGA). The differentially methylated genes were identified and protein–protein interacting network was constructed, with which prognostic signature of this cancer was generated. Survival and functional pathways analysis w used to evaluate the clustering ability of the prognostic signature.

**Results:**

We identified 151 differentially methylated genes related to relapse-free survival of patients with LUAD. Nine hub genes were identified in PPI network, with which 4 gene pair signature was selected as prognostic signature. The potential functions of 6 genes (JDP2, SERPINA5, PLG, SEMG2, RFX5, and POLR3B) in the 4-gene pair signature were enriched in intracellular protein synthesis and transportation.

**Conclusion:**

The four gene pair signature can predict the prognosis of patients with stage I LUAD. Our study provides a reference for patients with postoperative adjuvant therapy.

## Background

Lung cancer has two main types known as small-cell lung carcinoma (SCLC) and non-small-cell lung carcinoma (NSCLC) [1, 2]. Currently, no treatment can cure this kind of cancer, making lung cancer the leading cause of the death from cancer worldwide. Hereinto, lung adenocarcinoma (LUAD) is the main subtype of non-small cell lung cancer (NSCLC). With an increasingly high incidence, LUAD is always a threat to human beings regardless of gender and smoking condition over the past few decades in many countries [3]. Previous reports showed that patients with LUAD had a shorter survival time among patients with other types of non-small cell lung cancer (NSCLC) [4–6].

As a common histological subtype of NSCLC, most of clinical treatments of LUAD are basing on the cancer staging system of American Joint Committee on Cancer (AJCC) [7, 8]. Surgical resection is usually used to treat patients with stage ILUAD, but, achieved a poor prognosis. The recurrence rate of postoperative patients is as high as 35–50% [9]. For the large amount of patients suffering for reoccurrence, adjuvant chemotherapy could be an effective approach to significantly improve their survival time [10]. Thus, it is indeed urgent to discover an accurate and reliable clinical method to evaluate and/or predict the prognosis of LUAD, especially at early stage.

The most popular approach in recent years is to screen oncogenes or/and microRNAs as biomarkers for clinical diagnosis and treatment [11]. For diagnosis and treatment, the application of biomarkers can improve the survival of patients following with more personalized treatments [12, 13]. In addition to the genetic causes, of cancer, epigenetic changes also contribute to the development of cancer. Hereinto, DNA methylation is a kind of mark in a variety of tumors including lung cancer [14]. In DNA methylation, hypermethylation is recognized not only able to repress transcription of tumor suppressor genes, but also to trigger oncogenesis. It has been studied that tumor-suppressor genes are inactivated by the hypermethylations occurring oncytosine–guanine (CpG) island which locates in the promoter regions [15]. In addition, the enlarging public database for DNA methylation also provide wider and sufficient resources to study the mechanism and to explore methylated gene biomarkers [16, 17]. Taken these, identifying the target genes that can be silenced by DNA methylation or screen the cancer related methylations have a great impact on early diagnosis, cancer staging, and prognosis of cancer patients. However, less study is focusing on the relationships of DNA methylation and recurrence of LUAD patients [18].

Here selected the Cancer Genome Atlas (TCGA) database and downloaded methylated DNA data of LUAD patients. Then we identified 151 differentially methylated genes related to relapse-free survival (RFS) of patients with LUAD. Nine hub genes were identified in PPI network, with which 4 gene pair signature was selected as prognostic signature, which successfully clustered LUAD patients at stage I into high- and low-risk groups with significant differences. The potential functions of 6 genes (JDP2, SERPINA5, PLG, SEMG2, RFX5, and POLR3B) in the 4-gene pair signature were enriched in intracellular protein synthesis and transportation. The prognostic signature generated in the present study can predict the prognosis of patients with stage I LUAD, providing reference on the recurrence risk. The application of this signature will as well contribute to improve the overall survival of LUAD patients with postoperative adjuvant therapy. It should be noted that the signatures identified in this study are generally applicable. But for the personalized cases, specific methods would be applied in practice.

## Materials and methods

### Data source

Expressing profiles of gene-specific DNA methylation data and the follow-ups of corresponding patients were downloaded from database The Cancer Genome Atlas (TCGA, https://tcga-data.nci.nih.gov/tcga/) before April 17th, 2017. The clinic information of the patients was listed in detail (Table 2). The DNA methylation data was measured with Illumina Human Methylation 27 Beadchip (27K array) and Illumina Human Methylation 450 Beadchip (450K array). We focused on analyzing the 25,978 CpG sites measured by both 27K and 450K arrays. The probes were annotated to genes according to the annotation table of 27K platform.

### Data preprocessing and differentially methylated sites

To clear the DNA methylation data, CPG sites would be deleted if the CPGs were in absence in more than 50% of the total samples. CPG sites deficient in less than 50% samples would be weighted by using k-Nearest-Neighbor values [19].

Methylated CPG sites differentially expressed in samples with LUAD was screened by using SAM [20]. This process was repeated 1000 times for each disturbance. For the multiple binomial tests, the *p*-values are adjusted by the Benjamin and Hochberg method to control the false discovery rate (FDR) [21].

### Protein and protein interactions (PPIs)

To analyze the biological functions of the selected genes, protein–protein interacting (PPI) network was constructed to screen the hub genes associating with prognosis of LUAD [22]. All the protein networks used in the present study were obtained in database signor [23].

### Survival analysis

The correlation between each differentially methylated gene pair and the RFS was evaluated in univariate survival analysis. To estimate the independent prognostic value of this signature basing on several clinical factors including age, gender and smoking, the multivariate survival analysis was performed subsequently. Cox proportional-hazards regression model was applied in all the above survival analysis [24]. At the same time, the significance of the correlation between the ridge expansion osteotomy (REO) of a gene pair and the poor RFS was validated. Survival curves were drown by using the Kaplan–Meier method and were compared in the log-rank test [25].

### Enrichment analysis

The KEGG (Kyoto Encyclopedia of Genes and Genomes) pathways of selected methylated genes were analyzed by using DAVID (The Database for Annotation, Visualization and Integrated Discovery) [26]. Functional key genes were determined by Spearman’s correlation coefficient FDR < 0.01, r > 0.6.

## Results

### Data source

All gene-specific DNA methylation data were obtained from 578 patient samples from lung adenocarcinoma (LUAD), including 56 normal samples next to cancer tissues. There were 191 survival information containing RFS from 578 patients with stage I LUAD.

### Differentially methylated sites

In identification of the differentially methylated CPG sites (1000 times, FDR < 0.01), a total of 5029 CPG sites were identified up-regulated and 3269 ones were down-regulated. Then these differentially methylated CPG sites were mapped onto corresponding genes.

One gene would be defined as differentially methylated if one differentially methylated CPG site occurred on its promoter. On the contrary, if there were two methylated sites occurred on the promoter with different directions, this gene would be deleted. Finally, we obtained 3498 hypermethylation of genes and 2465 ultra-low methylated genes. In univariate Cox analysis basing on survival information, genes related to non-recurrence of postoperative patients with stage I LUAD were screened as well (p < 0.01), 61 hypermethylation and 90 ultra-low methylated genes.

### Hub genes screening in PPI network

**Fig. 1:**
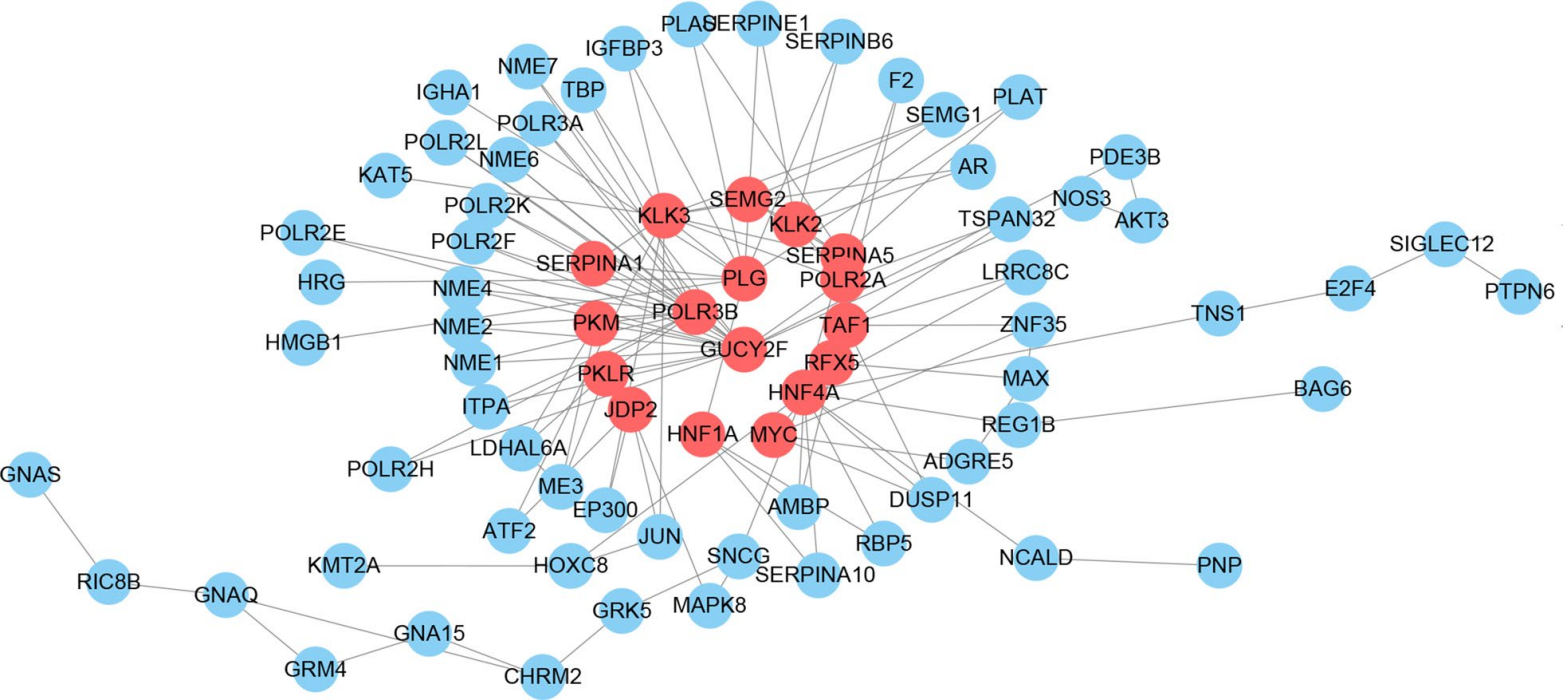
PPI network based on the survival 151 genes. Node represents the gene in the PPI network. Red represents the degree of the gene is over 3; blue represents the degree of the gene less than 3

With these 151 genes associating with non-recurrence of postoperative patients, PPI networks were constructed to identify hub genes. Totally, 17hub genes were obtained, indicating 9 key genes (KLK3, GUCY2F, KLK2, SERPINA5, PLG, SEMG2, RFX5, POLR3B, JDP2) relating to non-recurrence of postoperative patients with LUAD (Fig. 1).

**Fig. 2:**
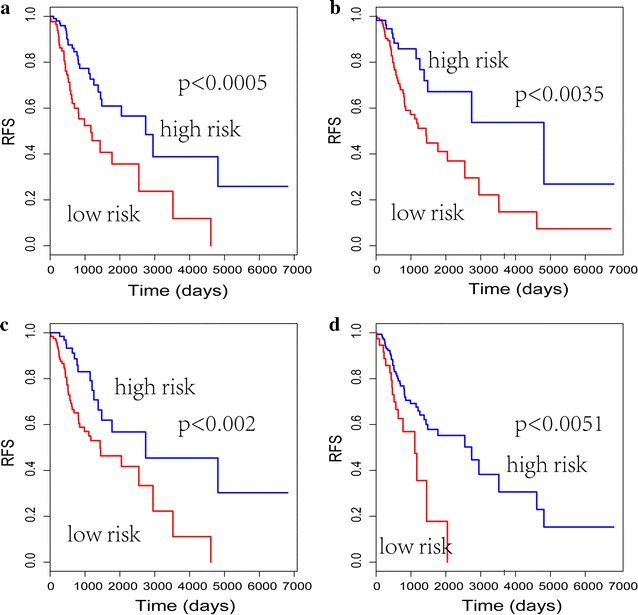
The association between four gene-pair signature and the RFS of 191 LUAD patients. The log rank p value is displayed separately. **a** High- and low-risk group clustered by gene pair 1; **b** high- and low-risk group clustered by gene pair 2; **c** high- and low-risk group clustered by gene pair 3; **d** high- and low-risk group clustered by gene pair 4

Survival-associated gene pairs were generated with these 9 key genes. Five gene pairs were identified significantly associating with survival of LUAD (p < 0.01) in univariate Cox analysis. By using the C-index of gene pairs and forward-stepwise algorithm, a 4-gene pair prognostic signature, consisting of 6 genes (Table 1), were generated for postoperative patients with stage I LUAD (Fig. 2). Postoperative patients with on gene pair of the 4-gene pair signature were defined as poor prognosis of recurrence. In Fig. 2, patients with stage I LUAD were able to be classified into high- and low-risk group with significant RFS.

**Table 1:** The composition of the signature

Signature	RMOs (Ma > Mb)^a^		Hazard ratio	*p* value
Gene pair 1	JDP2	SERPINA5	2.4315	0.0005
Gene pair 2	JDP2	PLG	2.4974	0.0035
Gene pair 3	JDP2	SEMG2	2.5136	0.0020
Gene pair 4	RFX5	POLR3B	2.2728	0.0051

^a^Represents the relative methylation ordering (RMOs) of gene pair (Ma > Mb), Ma and Mb represent the methylation value of genes a and b, respectively

### Survival analysis

**Fig. 3:**
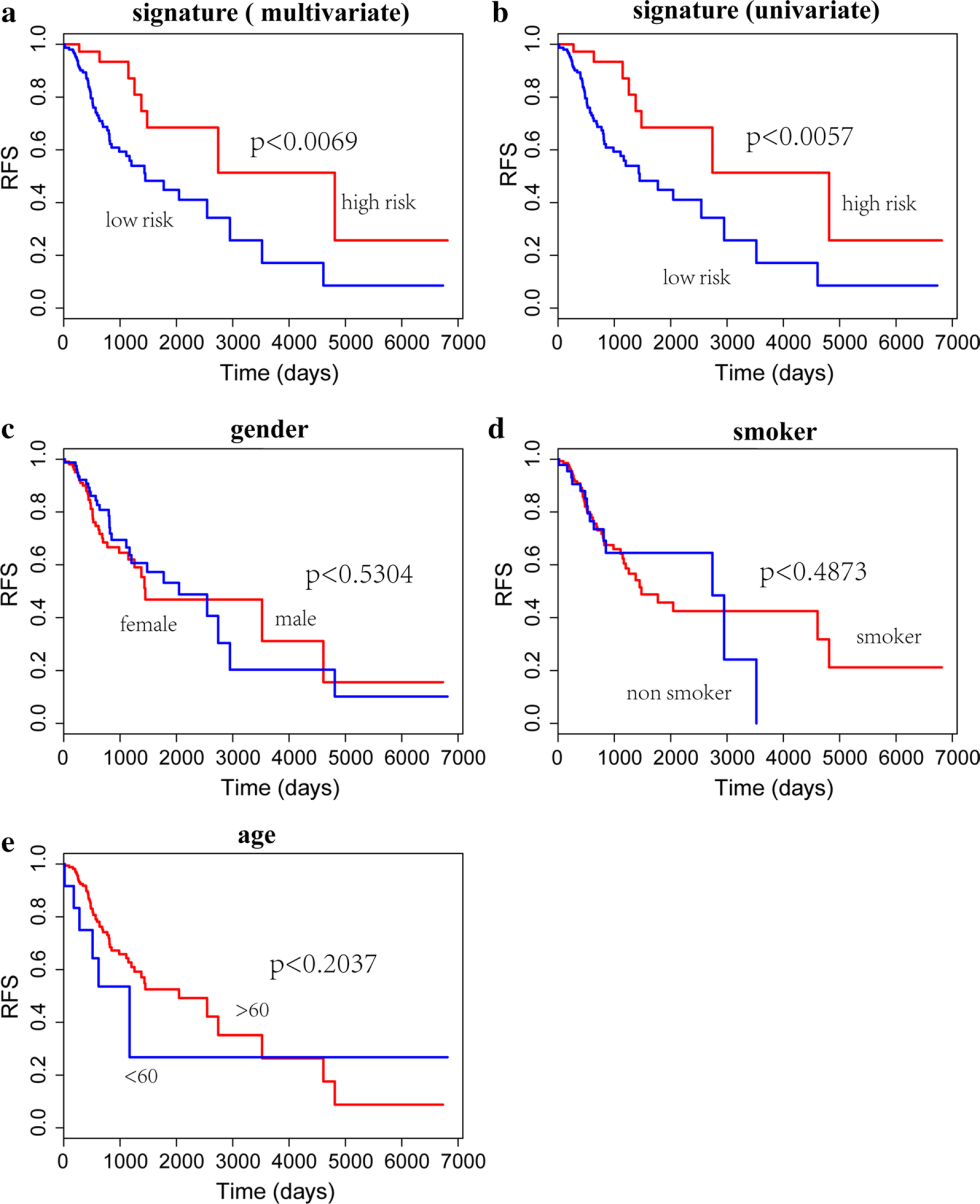
Survival analysis of 4 gene-pair signature. This signature was able to classify the stage I LUAD patients into two groups with significant RFS (univariate COX p < 0.0057, multivariate COX p < 0.0069). **a** Multivariate survival analysis of the signature; **b** univariate survival analysis of the signature; **c** survival analysis on the impacts of gender; **d** survival analysis on the impacts of smoking; **e** survival analysis on the impacts of age. The log rank p value is displayed separately

We applied Kaplan–Meier method and log-rank test to evaluate the impacts of prognostic signature casting on the RFS of stage I LUAD patients. The relationships of RFS time with age, gender and smoking condition were further analyzed (Table 2). Figure 3 indicated that the 4 gene-pair signature was able to classify the stage I LUAD patients into two groups with significant RFS (univariate COX p < 0.0057, multivariate COX p < 0.0069). However, in the relationships of RFS and age, gender and smoking condition, no factors showed influences on our prognostic signature.

**Table 2:** The clinical data for the 191 early stage LUAD

Covariates	Category	Total
Age years^a^	< 60	54
	≥ 60	131
Gender	Male	83
	Female	108
RFS status	Relapse	61
	Non-relapse	130
Smoker^a^	Non-smoker	141
	Smoker	46

^a^Information is partially lacked

### Enrichment analysis

The potential functions of 6 genes (JDP2, SERPINA5, PLG, SEMG2, RFX5, and POLR3B) in the 4-gene pair signature were further analyzed in enrichment analysis together with all 5963 differentially methylated genes. A total of 560 hypermethylation genes were screened according to the threshold of FDR < 0.01 and r > 0.6. In GO term, all these genes were associated with intracellular protein synthesis and transportation. All the enriched pathways were listed in Table 3.

**Table 3:** Enriched pathways of methylated genes in signature

Pathway name	p-value
Cellular protein localization	5.47E−07
Cellular macromolecule localization	6.42E−07
Intracellular protein transport	8.81E−07
Intracellular transport	4.37E−05
Macromolecule localization	5.02E−05
Cellular protein metabolic process	7.15E−05
Protein localization	0.000123001
Cellular localization	0.000128524
Protein transport	0.000204877
Negative regulation of cellular macromolecule biosynthetic process	0.000244491
Primary metabolic process	0.000251601
Protein metabolic process	0.000260279
Negative regulation of nucleobase nucleoside nucleotide and nucleic acid metabolic process	0.000341604
Cellular macromolecule metabolic process	0.000350947
Establishment of protein localization	0.000404266
Negative regulation of nitrogen compound metabolic process	0.000458234
Negative regulation of biosynthetic process	0.000512494
Protein modification process	0.000516455
Negative regulation of macromolecule biosynthetic process	0.000562752
Negative regulation of cellular biosynthetic process	0.000615668
Protein targeting	0.000632614
Establishment of localization in cell	0.000691823

## Discussion

In non-small cell lung cancer (NSCLC), lung adenocarcinoma (LUAD) is a common histological subtype with high mortality but poor outcome in both male and female, smokers and non-smokers [27]. To date, the current therapy of stage I LUAD patients is still surgery. However, almost half of the patients after surgical treatment suffer from recurrence or even death, resulting in a low 5-year survival rate [28, 29]. Therefore, an effective prognostic signature to evaluate the survival outcomes or to predict the risk of postoperative recurrence is in need.

In the present research, we screened out 560 hypermethylation genes (FDR < 0.01 and r > 0.6). But for further construction of prognostic signature, 151 genes associating with non-recurrence of postoperative patients were used to analyze for hub genes. Finally, 17 hub genes were obtained, indicating 9 key genes (KLK3, GUCY2F, KLK2, SERPINA5, PLG, SEMG2, RFX5, POLR3B, JDP2) relating to non-recurrence of postoperative patients with LUAD.In the selecting process of prognostic methylated genes, threshold of differential significance p < 0.01 was referred in univariate Cox analysis. In the present research, we generated a 4-gene-pair signature consisting of 6 genes (JDP2, SERPINA5, PLG, SEMG2, RFX5 and POLR3B) with potential functions in LUAD. All these genes were enriched in intracellular protein synthesis and transportation pathways in GO term. However, further functional analysis specifically on every gene was in need to explore the mechanism of this prognostic signature.

Subsequently, the signature was applied to evaluate the impacts of prognostic signature casting on the RFS of stage I LUAD patients and to detect the relationships between RFS and age, gender and smoking condition. It is satisfied that the 4 gene-pair signature was able to classify the stage I LUAD patients into two groups with significant RFS (univariate COX p < 0.0057, multivariate COX p < 0.0069). Meanwhile, the clustering ability of this signature was not influence by the age, gender and smoking condition. All these indicated that this signature was reliable and stable for evaluation of the prognosis of LUAD.

To get further knowledge of the 6 hub genes involved in the prognostic signature, we carried out literature review to explore the functions or association in cancer. Among these 6 genes, several were identified related to cancer. That is, most of the genes in the present study were firstly identified related to lung cancer.

Gene *JDP2* encodes Jun dimerization protein-2 that functions as AP-1 transcription factor. This protein can repress the transactivation mediated by the Jun family of proteins [30]. It has been identified that decreased-expression of JDP2 was related to lymph node metastasis and distant metastasis. Besides, its depression was as well found casting strongly relationships with the post-surgery survival time. Yuanhong et al. stated that there might be a possible relationship between the expression of JDP2 and metastasis in pancreatic carcinoma, suggesting JDP2 as a prognostic biomarker for patients with pancreatic carcinoma [31]. Besides, research also discovered an association of epithelial-mesenchymal transition (EMT) with Jun dimerization protein 2 (JDP2) in pancreatic cancer [32]. Hereinto, EMT is considered to contribute to the invasion and metastasis of a variety of malignant tumors. Taken all these previous studies which conform to our discovery, we suggest the JDP2 might play an important role in invasion and metastasis of LUAD as well. The signature model containing JDP2 is reasonable when clustering the LUAD patients. Further research focusing on the specific functions of this gene in lung is still in need to complete the mechanism of the recurrence of LUAD at gene level. Besides, the probable cooperating genes of JDP2 also deserve more attention.

Another cancer related gene discovered in our prognostic signature is SERPINA5, also called Protein C Inhibitor(PCI), belongs to the serine protease inhibitor super family as well. This gene is known to prevent metastasis and anti-angiogenesis in tumor cells, including renal, breast, prostate and ovarian cancers [33–37]. However, little research indentified this depression of SERPINA5 in LUAD till now. The association between SERPINA5 and this subtype of lung cancer is an inspiring result, but the expression status (at transcriptional or post-transcriptional level) and biological function of this gene in LUAD are largely unknown.

Semenogelin-2 (SEMG2) was ever found being catalyzed to degrade by the proteolytic activity of the active PSA-enzyme in prostate cancer [38] and that the functions in HER2+ cellular models of breast cancer [39]. As to RFX5 (Regulatory Factor X), being able to bind DNA and lend promoter specificity [40], was identified as a transcriptional activator of the TPP1 gene in hepatocellular carcinoma [41]. Besides, RFX5 was tested to express differentially in breast cancer [42]. POLR3B (Polymerase RNA III beta subunit) is reported to code for the other subunit that forms Pol III’s catalytic site, which transcribes small untranslated RNAs [43]. Researchers were searching the mutations in POLR3, so that its functions in several tumors could be revealed at mRNA levels by using RNA-Seq analysis [44]. Thus, the identification of this gene in LUAD might provide a reference for further studies.

According to the discussions above, we supposed that the 4-gene-pair signature could be used to evaluate the prognosis of LUAD patients generally. However, considering the gene diversity of cancer patients and even within a pedigree, personalized insight is also the key point for cancer managements including the prognosis and therapy.

## Conclusion

All in all, in the present study, we generated a 4-gene pair signature with 6 methylated genes. The signature was able to classify the stage I LUAD patients into two groups with significant RFS. However, age, gender and smoking condition did not influence the prediction of our prognostic signature. The potential functions of 6 genes were associated with intracellular protein synthesis and transportation. This signature might be used to provide clinical reference for postoperative chemotherapy of patients with stage I LUAD. In addition, our signature may independently predict the prognosis of LUAD without depending on data standardization.
